# Scaling and relations of morphology with locomotor kinematics in the sidewinder rattlesnake *Crotalus cerastes*

**DOI:** 10.1242/jeb.243817

**Published:** 2022-04-19

**Authors:** Jessica L. Tingle, Brian M. Sherman, Theodore Garland

**Affiliations:** Department of Evolution, Ecology, and Organismal Biology, University of California, Riverside, Riverside, CA 92521, USA

**Keywords:** Allometry, Biomechanics, Body size, Individual variation, Locomotion, Squamates

## Abstract

The movement of limbless terrestrial animals differs fundamentally from that of limbed animals, yet few scaling studies of their locomotor kinematics and morphology are available. We examined scaling and relations of morphology and locomotion in sidewinder rattlesnakes (*Crotalus cerastes*). During sidewinding locomotion, a snake lifts sections of its body up and forward while other sections maintain static ground contact. We used high-speed video to quantify whole-animal speed and acceleration; the height to which body sections are lifted; and the frequency, wavelength, amplitude and skew angle (degree of tilting) of the body wave. Kinematic variables were not sexually dimorphic, and most did not deviate from isometry, except wave amplitude. Larger sidewinders were not faster, contrary to many results from limbed terrestrial animals. Free from the need to maintain dynamic similarity (because their locomotion is dominated by friction rather than inertia), limbless species may have greater freedom to modulate speed independently of body size. Path analysis supported: (1) a hypothesized relationship between body width and wavelength, indicating that stouter sidewinders form looser curves; (2) a strong relationship between cycle frequency and whole-animal speed; and (3) weaker effects of wavelength (positive) and amplitude (negative) on speed. We suggest that sidewinding snakes may face a limit on stride length (to which amplitude and wavelength both contribute), beyond which they sacrifice stability. Thus, increasing frequency may be the best way to increase speed. Finally, frequency and skew angle were correlated, a result that deserves future study from the standpoint of both kinematics and physiology.

## Abstract (precis)

**Summary:** Examination of ontogenetic scaling of morphology and locomotion in sidewinder rattlesnakes provides evidence for several significant relationships among morphology, kinematics and whole-animal speed.

## INTRODUCTION

Previous studies of terrestrial locomotion have demonstrated how aspects of kinematics scale with body size inter- and intraspecifically for walking, running and jumping (e.g. Day and Jayne, 2007; Emerson, 1978; Heglund et al., 1974; Irschick and Jayne, 2000; Pennycuick, 1975; Smith et al., 2010; Toro et al., 2003). However, many terrestrial animals navigate the world without limbs, and they face different locomotor challenges compared with limbed animals. A limbless body plan has evolved more than 25 times in terrestrial vertebrates and represents 19% of terrestrial vertebrate diversity (∼4300 species) (Astley, 2020; Bergmann et al., 2020; Wiens et al., 2006). Of the limbless terrestrial vertebrates, which include caecilians and numerous squamate reptiles, no clade surpasses snakes in their locomotor diversity. Slithering, crawling, climbing and even gliding snakes manage a remarkable variety of motions (Jayne, 2020).

Limbless terrestrial animals differ from limbed ones in fundamental ways that likely influence the scaling of kinematics during locomotion. For example, limbed terrestrial animals face high postural costs at large body sizes because mass increases with length cubed while limb cross-sectional area increases with only length squared. To deal with the disproportionate demands of locomotion at larger body sizes, they may evolve morphological ‘solutions’, such as thicker limbs, they may alter their behavior in the gross sense (such as avoiding especially taxing tasks like jumping or climbing) and/or they may alter kinematics parameters, such as posture (sprawling versus upright) or duty factor (e.g. Biewener, 1989; Cieri et al., 2021; Day and Jayne, 2007; Hutchinson et al., 2006). In contrast, limbless animals usually keep their bodies largely in contact with the ground, so one would expect them to incur lower postural costs, even at relatively large body sizes.

Moreover, the forces involved in limbless versus limbed locomotion differ substantially. Limbed locomotion is dominated by inertial forces and can be understood through the lens of dynamic similarity. If a motion scales with dynamic similarity, then geometric and temporal variables scale in such a way that the ratio between dominant forces remains constant (e.g. Froude number of walking or running animals, which equals the ratio of centripetal to gravitational force) (Alexander, 1991; Alexander and Jayes, 1983). In contrast, limbless terrestrial locomotion is dominated by friction rather than inertia; one study estimated frictional forces to be more than 10-fold greater than inertial forces in snakes moving via lateral undulation (Hu et al., 2009). The moment a snake stops exerting force on its environment, it stops moving, much like a tiny organism in a viscous fluid (Vogel, 2003). This fundamental difference from walking, running and other limbed gaits means that dynamic similarity does not apply.

**Fig. 1.**
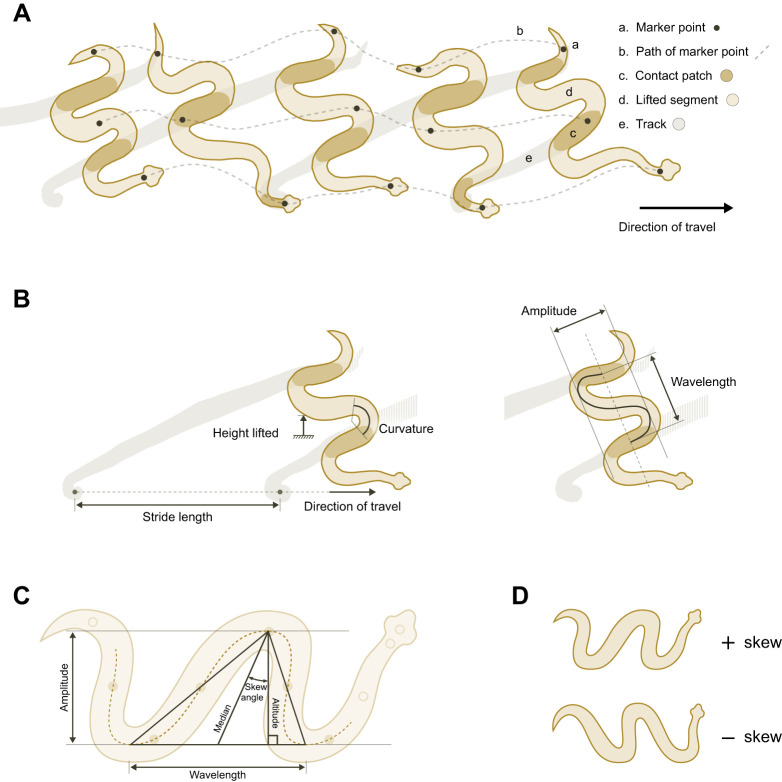
**Kinematics of sidewinding movements in sidewinder rattlesnakes.** (A) Sidewinding snakes move in a direction oblique to their body axis, propagating waves that have a horizontal as well as a vertical component. At any given time, some sections of the body remain in static contact with the ground while other sections are lifted up and forward to a new contact patch. (B) The shape of a sidewinder's body can be described using common wave properties, including peak-to-peak amplitude and wavelength. Stride length is the distance between successive tracks in the direction of travel. Because the body axis is oblique to the direction of travel, both amplitude and wavelength contribute to stride length and their relative contributions are determined by other aspects of the wave's shape, such as skew angle. (C) Wavelength is the distance between successive maxima (crests) or successive minima (troughs). If we draw a triangle between two minima and the maximum in between them (or two maxima and the minimum in between them), then skew angle is the angle between the triangle's median and any line perpendicular to the line connecting the minima (or the maxima). Amplitude is the triangle's altitude, which equals the median times the cosine of the skew angle. (D) Positive skew angle indicates that waves are tilted towards the head, whereas negative skew angle indicates a tail-wards tilt. A and B are traces from high-speed video of *Crotalus cerastes*, modified with permission from Tingle (2020). C and D are stylized drawings.

The present study focuses on a type of locomotion called sidewinding, which is best known in several viper species from sandy desert environments. Sidewinding snakes move in a direction oblique to the axis of their bodies, propagating waves that have a horizontal as well as a vertical component. At any given time, some sections of the body remain in static contact with the ground while others are lifted up and forward to a new contact patch (Fig. 1A). Several aspects of sidewinding have received attention (see Tingle, 2020 for a review), including general descriptions of kinematics (e.g. Gans and Kim, 1992; Gray, 1946; Jayne, 1986) and mechanisms for specific tasks such as ascending slopes (Marvi et al., 2014), turning (Astley et al., 2015) and negotiating obstacles (Astley et al., 2020b). One study dealt with the scaling of sidewinding performance (burst speed and endurance) (Secor et al., 1992), but none has focused on the scaling of sidewinding kinematics, despite the ubiquity of scaling effects on other types of locomotion (Cloyed et al., 2021; Garland and Albuquerque, 2017; Pedley, 1977).

Robots can imitate the kinematics of sidewinding snakes even though they generally move more slowly than the snakes do (e.g. Astley et al., 2015; Gong et al., 2014; Marvi et al., 2014), providing evidence that sidewinding, like lateral undulation, is dominated by friction rather than inertia. Additionally, a theoretical approach using geometric mechanics (Astley et al., 2020a; Rieser et al., 2019 preprint) models sidewinding with high accuracy, despite its inability to account for inertial effects. Therefore, we might predict geometric similarity for linear dimensions describing the shape of the wave made by the body, such as wavelength, amplitude and the height of vertical lifting (Fig. 1B); that is, these dimensions would have an expected scaling exponent of 1 against snout–vent length. In addition to simple linear dimensions, the waveform of a sidewinder's body can vary in the degree to which it tilts towards either the head or the tail, which we call the skew angle (Fig. 1C,D). Skew angle has not previously been considered, but it might be expected not to vary systematically with body size under geometric similarity because sidewinders of different sizes should have the same wave shape.

For the frequency of a sidewinding cycle, it is more difficult to predict scaling. On one hand, frequency generally decreases with body size for locomotion involving oscillation, such as flapping flight, swimming via tail beats, and running, and this relationship exists due to physical laws and the intrinsic properties of muscles (e.g. Bainbridge, 1958; Drucker and Jensen, 1996; Heglund and Taylor, 1988; Norberg and Norberg, 2012; Rayner, 1988; Smith et al., 2010). On the other hand, sidewinding involves a travelling wave and so is qualitatively different from these locomotor modes. Richard and Wainwright (1995) used a simple mechanical model of the musculoskeletal anatomy as a lever system and incorporated unloaded muscle contraction velocities, which led them to predict that gape cycle time (and therefore frequency) of feeding fish would not change with body size. Their approach could also have utility for locomotor kinematics. We could also formulate a hypothesis for the scaling of frequency on a geometric or general comparative basis. Following the arguments presented in a seminal paper by Hill (1950), we might reasonably predict that whole-animal speed and acceleration would not change with body size. In that case, as sidewinders increase in size, they would move with lower frequency, such that their speed would not change even if they travel a greater distance during each cycle of sidewinding. However, it would also not be surprising for larger sidewinders to move with the same frequency as small ones and to achieve higher speeds, given that intraspecific analyses of a variety of animals indicate that routine and maximal speeds often increase with size (Cloyed et al., 2021). Finally, acceleration should not change with body size based on the mathematical model of Cloyed et al. (2021).

After accounting for body size, morphological variation may lead to kinematic and performance variation. Previous studies have shown that sidewinding viper species have some morphological specializations (Jayne, 1982; Rieser et al., 2021; Tingle et al., 2017; but see Tingle and Garland, 2021); however, none has explored the link between morphology and sidewinding locomotion at the intraspecific level. Sidewinding snakes form curves along the body (Fig. 1B), and a snake's maximum potential curvature might depend on such morphological traits as body width and number of vertebrae (both of which vary intraspecifically) (Brainerd and Patek, 1998; Moon, 1999; Morinaga and Bergmann, 2019; Sharpe et al., 2015). Additionally, the tail does not seem to contribute to force production during sidewinding (Jayne, 1988), so relatively long tails may inhibit performance, for example by reducing the frequency of sidewinding cycles. Note, however, that any relationship between tail length and locomotion may be complicated by the use of tails for signaling or other non-locomotor behaviors in some species, such as rattlesnakes.

The contributions of various kinematic parameters to performance, as well as the relationships among kinematic parameters, also merit further exploration to improve our mechanistic understanding of sidewinding. For example, we do not currently know the degree to which various wave shape parameters contribute to stride length, which refers to distance travelled per cycle, and has been used to characterize not only walking and running, but also such locomotor modes as swimming (e.g. Aubret and Shine, 2008; Clark and Bemis, 1979; Drucker and Jensen, 1996; Svendsen et al., 2016; Videler and Wardle, 1991; Wardle, 1975) and crawling (Berrigan and Pepin, 1995; Quillin, 1999).

Here, we use morphometric and high-speed video data to examine factors influencing sidewinding locomotion in the sidewinder rattlesnake *Crotalus cerastes*. We first explore the effects of size, sex and age class (juvenile versus adult) on morphology and kinematics. Then, we use path analysis to test hypothesized causal relationships and correlations among morphological, kinematic and speed. Our hypotheses included the following: (1) frequency as well as any wave parameter contributing to stride length might show a causal relationship with snakes' overall speed; (2) vertebral count, mass and/or body width relative to snout–vent length might show a causal relationship with any kinematic variable associated with curvature (wavelength and/or amplitude, in our dataset); and (3) longer tails might reduce the frequency of the motion. Additionally, we expected that we might find negative correlations between frequency and wave parameters that increase stride length, which would indicate a trade-off. Finally, snakes are not infinitely long, so we anticipated the possibility of negative correlations between kinematic variables that require snakes to expend part of their body length (wavelength, amplitude and height lifted).

## MATERIALS AND METHODS

### Data collection

We collected sidewinder rattlesnakes (*Crotalus cerastes* Hallowell 1854) on the Barry M. Goldwater Range near Yuma, Arizona, USA in June and July 2016. Our sample included 74 female and male snakes ranging from small juveniles (young of the year, as determined by size) up to large adults. Research procedures were approved by the San Diego State Institutional Animal Care and Use Committee (permit number 16-08-014C).

We anesthetized snakes by placing them in a tube with a cotton ball soaked in approximately 1 ml isoflurane per 500 g of snake mass (never <0.125 ml isoflurane). While the snakes were anesthetized, we determined sex by cloacal probing and collected the following measurements: mass (to 1–5 g of accuracy with Pesola scales or a digital scale); snout–vent length (SVL) and tail length (both to the nearest mm with measuring tape); width at 25%, 50% and 75% of the SVL (to the nearest mm with calipers); neck width, head width at the corners of the mouth and head length from the anterior edge of the first ventral scale (to the nearest mm with calipers); number of ventral scales (following the convention of Dowling, 1951); number of subcaudal scales; and number of dorsal scale rows. Table 1 lists all of the morphometric and meristic traits that were measured. Finally, we painted 10 markers along the dorsum with White-Out brand correction fluid and black permanent marker as a visualization aid for the videos. Marker 1 was painted on the head between the eyes, marker 2 on the neck, markers 3–8 approximately evenly spaced along the body, marker 9 just above the cloaca and marker 10 at the end of the tail just before the rattle.

**Table 1.**
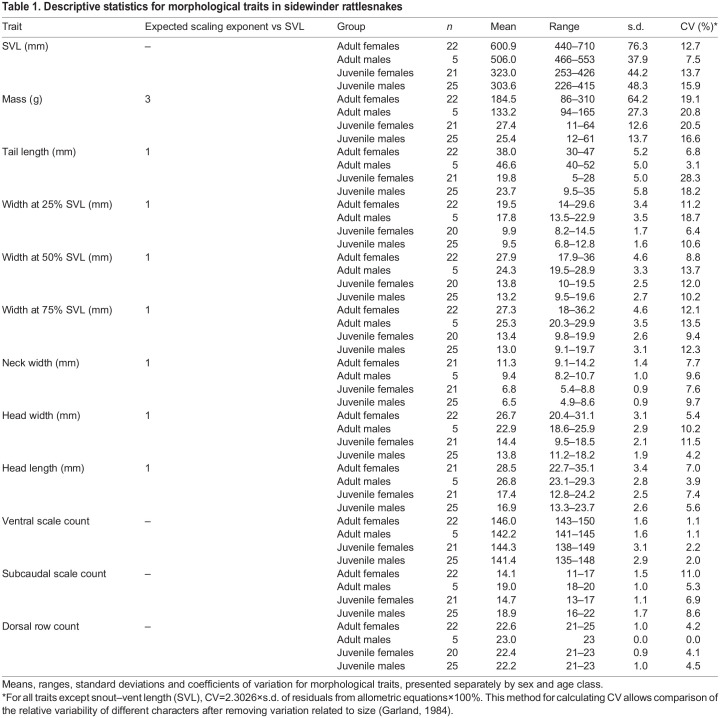
Descriptive statistics for morphological traits in sidewinder rattlesnakes

Approximately 1 day passed between the time of recovery from isoflurane and time of kinematics data collection. We recorded sidewinding sequences indoors in a sandbox measuring 1.15×1.15 m with two Edgertronic high-speed cameras (Model SC1; San Jose, CA, USA), synchronized at 500 frames s^−1^, with a resolution of 1264×1008 pixels. Cameras were placed ∼1.5–2 m away from the sandbox, with one camera on a low tripod for an oblique view that was as laterally oriented as possible, while the other camera was placed on a higher tripod for an oblique view that was as dorsally oriented as possible. In positioning the cameras, we attempted to include the entire sandbox in the field of view. Linear dimensions in videos were calibrated in the MATLAB program DLTdv5 (https://biomech.web.unc.edu/dltdv/) with a large object of known dimensions (several metal rods fixed to each other and to a metal base plate), which we placed in the middle of the sandbox. We recorded substrate and snake body temperatures for each trial. Substrate temperatures ranged from 20.4 to 27.2°C, while snake body temperatures ranged from 20.1 to 27.3°C, well within the active range observed in free-living sidewinders (Cowles and Bogert, 1944; Moore, 1978; Signore et al., 2022). Trials took place between 11:45 h and 23:28 h. Sand came from the Barry M. Goldwater Range about 14.5 km from where snakes were captured. Sand in the box measured 2 cm deep. We recorded sidewinding sequences that had at least 2–3 full cycles within the frame of recording. For each snake, we took three recordings. Snakes were given the minimum motivation necessary to elicit sidewinding; in some cases, it was enough to place them on the sandbox, whereas other cases required waving snake tongs, or tapping the tongs on either the substrate or the snake's tail. Note that this approach to motivating the snakes was not designed to elicit maximal performance (see Careau and Garland, 2012). In between trials, we raked and smoothed the sand to create a level surface.

### Video data pre-processing

We recorded trials for 66 individuals, aiming to obtain a final sample of 25–30 individuals for kinematics trials and knowing that not all trials would be usable. Of those, we chose to digitize videos based on a number of factors. First, we eliminated individuals that refused to perform multiple sidewinding cycles without stopping or turning, whose painted markers had rubbed off, or whose trials suffered from poor video or calibration quality (e.g. because a camera had been bumped). Of the remaining individuals, we chose ones that provided good representation from the total size range (evaluated based on both SVL and mass): the 3–5 largest females and males, the 3–5 smallest females and males, and several individuals of both sexes distributed throughout the middle of the size range. Our final digitized sample comprised 14 females and 12 males.

Because the raw videos were very large files, and 500 frames s^−1^ was more than adequate to quantify the motion, we converted the raw files from .mov to .mp4 format and then used Adobe Premiere to trim and downsample the videos, removing every other frame. Then we exported the trimmed videos as 30 frames s^−1^ mp4 files. We calibrated and digitized videos using the MATLAB programs DLTcal5 and DLTdv5 (https://biomech.web.unc.edu/dltdv/), which yielded files containing *x*, *y*, *z* coordinates of each tracked point at each frame.

We smoothed the data using a custom MATLAB program written by B.M.S. The program used a Savitzky–Golay filter (Savitzky and Golay, 1964), implemented by the built-in MATLAB function sgolayfilt. A Savitzky–Golay filter is an *n*th order moving regression. Like other smoothing algorithms, it functions as a low pass filter. This type of filter is effective for movement data as long as the filter is appropriately tuned (Crenna et al., 2021). Displacement was smoothed using a 3-pass fourth order Savitzky–Golay filter with a uniform weight distribution. Velocity and acceleration were computed from smoothed displacement using the finite difference method (first and second order central differences, respectively), and then smoothed using a single-pass fourth order Savitzky–Golay filter with a uniform weight distribution. In all cases (displacement, velocity and acceleration), we used a span of 143 frames in the smoothing functions. To eliminate edge effects, we dropped 150 frames at the beginning of each sequence and 100 frames at the end. This process produced smoothed displacement, velocity and acceleration for each of the 10 markers.

### Extracting kinematic variables

We used a custom MATLAB program written by B.M.S. to extract kinematic variables from the smoothed data. Some of these variables describe the whole snake's motion, some describe the motion of the 10 discrete markers painted on the body, and some describe the waveform of the snake's body. Table 2 lists all the kinematic variables that were quantified, along with scaling expectations under geometric similarity.

**Table 2.**
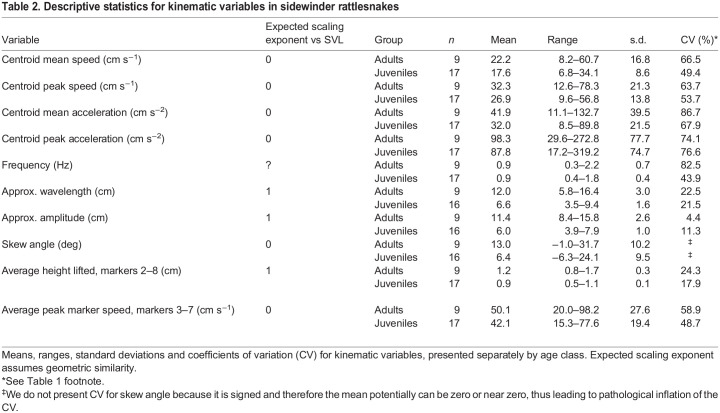
Descriptive statistics for kinematic variables in sidewinder rattlesnakes

To understand whole-snake speed and acceleration, we used the centroid of the 10 painted markers as the best approximation we could make for center of mass. First, we computed the displacement of the centroid at each frame using the smoothed displacements of the painted markers. Next, we calculated the velocity of the centroid in each frame using the central difference formula. Finally, we computed whole-snake speed from the velocity vector and took the average and peak speed over the whole trial. We calculated mean and peak centroid acceleration in a similar manner, using the second order central difference of centroid displacement.

For each of the 10 painted markers, we calculated peak speed (cm s^−1^) as well as maximum amplitude in the vertical direction (cm), i.e. the maximum height to which the marker was lifted over the course of the trial. We then used values from individual markers to calculate the mean value of those markers' peak speeds and heights lifted for a given trial. We did not use all 10 painted markers to calculate these mean values because we wanted to capture locomotor behavior, and the head and tail can be involved in non-locomotor behaviors (in these trials, non-locomotor behaviors included surveying the environment and rattling). Therefore, we needed to determine how many markers to discard from the head and tail regions. For each variable, we first replaced the raw data with *z* scores, which provide a sense of how far from the mean a data point is. *Z* (standardized) scores were calculated as:

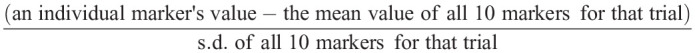


For each of the 10 markers, we calculated the mean *z* score across all trials for all individuals. We then determined which of the markers were most consistently close to the mean values (consistently had the lowest *z* scores). For peak speed, markers 3–7 were consistently closest to the trial mean, so we calculated an average value of peak speed for each trial based on those markers. For height lifted, we used markers 2–8.

Finally, to examine the body's waveform, we measured three common wave properties (frequency, wavelength and amplitude), plus skew angle, which describes the degree to which the wave slants towards either the head or the tail (Fig. 1). We calculated these based on painted markers 4–9 because the head/neck region (markers 1–3) and the tail (marker 10) moved less predictably than the rest of the body did, as explained above. For frequency, we used smoothed displacement data to measure the period of the wave for each sidewinding cycle, and then calculated the frequency as the reciprocal of the median period for the trial. With only 10 painted markers, we could not create a spline that accurately represented the shape of the body. However, the high temporal resolution of our data allowed us to estimate wavelength, amplitude and skew angle without reconstructing the snake's midline. To do so, we had to assume that snakes were moving at steady state, and that body shape of a sidewinding snake is a traveling wave where one mode dominates (i.e. most of the signal in the data results from the steady-state sidewinding motion). Each of the painted markers had to pass through the extrema of interest (the crests and troughs of the wave) at some point during a sidewinding cycle. When the angle formed by any three points was at a minimum, the middle point was assumed to be at an extreme (crest or trough). Given these times and locations of the extrema in a subset of frames, we estimated the locations of the extrema at all points in time using simple linear interpolation.

Wavelength is the distance between successive maxima (crests) or successive minima (troughs). If we draw a triangle whose corners are two minima and the maximum in between them (or two maxima and the minimum in between them), then the altitude of the triangle is the wave's peak to peak amplitude, and the angle between the altitude and the median is the skew angle (Fig. 1C). We calculated the median directly from our estimated extrema locations: one endpoint of the median is the midpoint of the line connecting the minima (or the maxima) and the other endpoint of the median is the maximum in between those two minima (or the minimum between the maxima). The skew angle is the angle between the median and any line perpendicular to the line connecting the minima (Fig. 1C). A positive skew angle indicates that the waves are tilted towards the head, while a negative skew angle indicates that the waves are tilted towards the tail (Fig. 1D). Note that sidewinding shows ‘handedness’, in that the snake's trunk can be positioned either to the left or the right of its head, and the program used to extract kinematics variables could not distinguish between left- and right-‘handed’ trials when determining the sign of skew angle. Therefore, we had to manually change the sign of skew angle for all left-handed trials prior to statistical analysis. The altitude/amplitude is the median times the cosine of skew angle (Fig. 1C). The reported values of wavelength, amplitude, and skew angle for each trial are the average over all points and frames where values could be calculated.

As the MATLAB program processed each trial, it displayed an animation of digitized points (using the smoothed displacements) and the interpolated wave extrema locations. This allowed us to qualitatively verify the extracted variables to check for anomalies, which can result from violations of the steady-state movement assumption (e.g. if a snake turned partway through a trial rather than proceeding along a relatively straight path). In cases where we detected anomalies, we either truncated the trial to omit the affected frames and re-analyzed it, or we discarded the trial entirely prior to statistical analysis. Our final sample included 63 total trials for 26 individuals; some of these trials were missing one or more variables because wave properties could not be calculated if a trial was too short.

### Statistical analysis

All statistical analyses were implemented in R 3.6.0 (https://www.r-project.org/) except where otherwise stated. We log_10_-transformed morphometric traits prior to analyses. We checked for outliers using standardized residuals obtained by regressing each trait on SVL+sex, for juveniles and adults separately. If a standardized residual exceeded ∼3 in magnitude and/or was >1 s.d. from the next value, then the individual snake was removed as a statistical outlier for all further analyses involving that trait. Tables 1 and 2 present descriptive statistics for morphological traits and kinematic variables, respectively, with outliers removed.

Our main analyses proceeded in three stages, described in detail in the following paragraphs. First, for each morphological and kinematic trait, we examined variation related to sex and age by comparing ANCOVA models containing different combinations of SVL, sex and age as predictors. Second, we calculated bivariate reduced major axis (RMA) and ordinary least squares (OLS) slopes to examine scaling relationships. For each trait, we divided the sample into the subgroups suggested by that trait's best ANCOVA model (i.e. we analyzed females and males separately if the best ANCOVA model included sex, juveniles and adults separately if the best ANCOVA model included age, and all possible sex by age categories separately if the best ANCOVA model included both sex and age). Finally, we used path analysis of residuals (from log–log regressions on SVL, sex and/or age, according to the ANCOVA results) to test relationships of morphology, kinematics and speed.

We used ANOVA to test for sex differences in SVL, examining juveniles and adults separately. We then used ANCOVA (package *car*; Fox and Weisberg, 2019) with Type III sums of squares to test for effects of SVL, sex and age class (juvenile versus adult) on most morphological traits. We excluded dorsal row count, which showed minimal variation, and ventral scale count, which violated the assumption of homogeneity of variance (Levene's test *F*_3,69_=4.071, *P*=0.010). For each trait, we started with a full model that included SVL+sex+age+SVL*sex+SVL*age+sex*age+SVL*sex*age, and we then eliminated predictor variables in a stepwise fashion, starting with interaction terms, to determine the best-fitting model for each trait based on AICc (Table S1). In two cases where two models had AICc values within 2 (width at 25% SVL and head length), we chose the model that included more predictor variables to facilitate a more granular view of scaling relationships that might differ among groups. Because ventral scale count varied among individuals but violated the assumptions of homogeneity of variance, we used ANCOVA to test for sexual dimorphism in juveniles and adults separately, with SVL as a covariate (package *car*; Fox and Weisberg, 2019). It did not show a statistically significant relationship with SVL in either age group, so we excluded it from analyses of scaling relationships.

To examine scaling, we separated the sample into the subgroups suggested by the best model for each trait and calculated both RMA and OLS slopes (R 3.6.0, package *lmodel2*; https://CRAN.R-project.org/package=lmodel2). Confidence intervals for RMA slopes were calculated in *lmodel*2 using the formula from Jolicoeur and Mosimann (1968), and we identified deviations from isometry by determining whether those confidence intervals contained the expected value under isometry (3 for mass; 1 for linear measurements).

For statistical analysis of kinematic data, we chose one representative trial for each individual. To determine which trials would serve as representatives, we first ruled out those with incomplete data (unless all trials for an individual had incomplete data, in which case we considered trials that had the least missing data). We then watched the remaining videos and ruled out any with obvious issues (e.g. part of the body out of frame or obscured from view during part of the video). Finally, we counted the number of sidewinding cycles in each of the remaining videos, and we chose the video that maximized the number of cycles and the path length (the video with the most cycles almost always had the longest path length).

We then compared ANCOVA models as for the morphometric traits, but for the kinematic variables we included an additional set of models with snake body temperature as a predictor (we did not consider interactions between body temperature and other predictors) (Tables S2 and S3). Based on these models, only three kinematic variables were significantly related to SVL: wavelength, amplitude and height lifted. For these variables, we tested for isometric versus allometric scaling using the best combination of predictor variables, as we did for morphometric traits.

To calculate relationships of morphometric and/or kinematic variables, we log transformed them (except for skew angle, which is signed), regressed each variable on log SVL (including sex and/or age class as predictors in the regression if they appeared in the best ANCOVA model for a given variable) and then used the residuals to compute Pearson correlation coefficients and to conduct a path analysis. Previously identified outliers were removed prior to computing residuals.

**Fig. 2.**
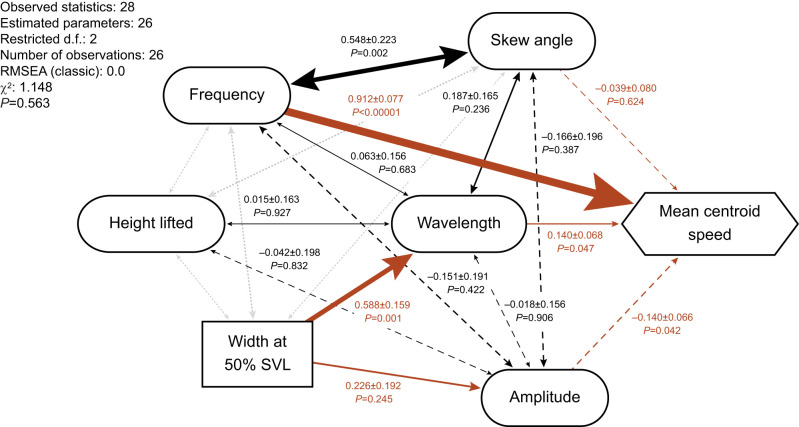
**Path model of hypothesized relationships among morphological traits, kinematic variables and speed.** Morphological traits (rectangles), kinematic variables (ovals) and speed (hexagon) and their causal relationships [by convention represented as one-headed arrows (here in red)] and correlations (two-headed arrows). Adjacent numbers are estimates and standard errors from Ωnyx (Onyx), in addition to *P*-values from likelihood ratio tests. Solid arrows correspond to positive estimates, whereas dashed arrows correspond to negative estimates. Correlations required for model fitting are shown as dotted gray two-headed arrows without estimates or *P*-values. The text in the upper left corner provides a summary of the model. RMSEA (root mean square error of approximation) and χ^2^ both provide an indication of model fit, and the *P*-value corresponds to the χ^2^ lack-of-fit test. In addition to this model, we considered several alternatives that included different morphological traits (tail length, mass and ventral scale/vertebral count), but they did not perform as well (see Materials and Methods and Table S5).

We conducted path analyses in Ωnyx (Onyx) (von Oertzen et al., 2015) to estimate parameters in a hypothesized causal model of relationships involving morphology, kinematics, and performance (Fig. 2). We used mean centroid speed as our measure of performance (but again, note that it does not represent maximal performance); we did not include additional measures of speed or acceleration because all measures of speed and acceleration were highly correlated (Table S4), which would lead to problems of multicollinearity. Speed equals frequency times stride length. Although we were unable to compute stride length from our data, we were able to compute some wave parameters that contribute to stride length. In sidewinders, stride length is determined in part by both wavelength and wave amplitude, as a result of the oblique angle between the sidewinder's direction of travel and the axis of the wave made by its body (Fig. 1C). The degree to which wavelength and amplitude contribute to stride length is determined in part by the wave's skew angle. Therefore, we hypothesized causal relationships of frequency, wavelength, amplitude and skew angle with mean centroid speed. We expected that we might find negative correlations between frequency and one or more variables that contribute to stride length, which would indicate a trade-off. We also included height lifted as an additional kinematic variable because the snake has to allocate part of its finite length to displacement in the vertical direction as well as in the horizontal plane, so height lifted, amplitude, and wavelength may therefore be correlated.

Additionally, we hypothesized that morphological traits affecting a snake's maximum potential body curvature may show causal relationships with amplitude and/or wavelength. One such trait is vertebral count (which is equal to ventral scale count). Another would be the stoutness of a snake's overall body shape, which could be described by mass and/or a width measurement. Finally, because the tail does not seem to contribute to force production during sidewinding (Jayne, 1988), we hypothesized that longer tails might inhibit sidewinding, reducing frequency.

Our sample size limited us to models with only seven total variables. Given that we identified ten potential variables of interest, we compared models with different combinations of those variables (Table S5). All models included hypothesized causal paths from frequency, wavelength and peak-to-peak amplitude to mean centroid speed, because we had strong reason to think that those variables would show the clearest relationships. In addition to those four variables, the models included all possible combinations of tail length, ventral scale count, mass and width at 50% SVL with their hypothesized effects on kinematics (except mass plus width at 50% SVL, which are redundant as measures of stoutness). We rejected nine of the 16 models because they had significant lack of fit based on a χ^2^ lack-of-fit test. We compared the remaining models using root mean square error of approximation (RMSEA), which measures lack of fit per degree of freedom (Browne and Cudeck, 1992; Rigdon, 1996). Lower values indicate closer fit, with a lower bound of zero. Six models had RMSEA of zero. Of the variables included in those six models, skew angle and body width consistently had strong relationships with other variables, whereas height lifted and mass did not (models with vertebral count always showed significant lack of fit). We therefore present the model that includes skew angle, body width and height lifted, which also had the lowest AICc of the six models with RMSEA of zero (Fig. 2).

## RESULTS

Juveniles did not show statistically significant sexual dimorphism for snout-vent length (*F*_1,44_=2.251, *P*=0.141), but adult females were longer than adult males (*F*_1,25_=7.231, *P*=0.0126). Sex was a significant predictor in the best ANCOVA models for tail length, head length, and subcaudal scale count, indicating sexual dimorphism in those traits (Table 3). The ANCOVA models also indicated SVL as a significant predictor for all morphological traits except scale counts. Additionally, age class was a significant predictor in the best models for mass, tail length, and head width. The interaction between SVL and age class was a significant predictor for tail length, indicating different scaling relationships in juveniles versus adults. The ANCOVAs for ventral scale count in juveniles and adults showed significantly higher values in females versus males in both age groups (*F*_1,43_=11.234, *P*=0.002 in juveniles; *F*_1,24_=18.499, *P*=0.0002 in adults), and no relationship between SVL and ventral scale count in either age group (*F*_1,43_=0.465, *P*=0.499 in juveniles; *F*_1,24_=0.023, *P*=0.881 in adults).

**Table 3.**
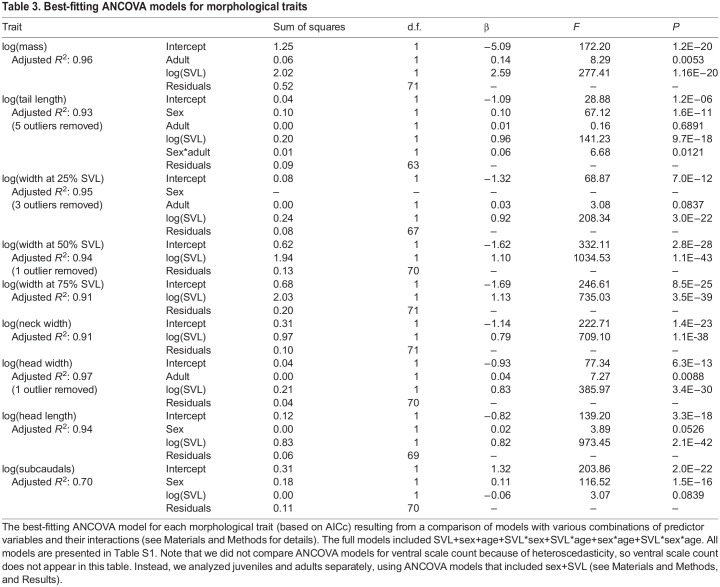
Best-fitting ANCOVA models for morphological traits

**Fig. 3.**
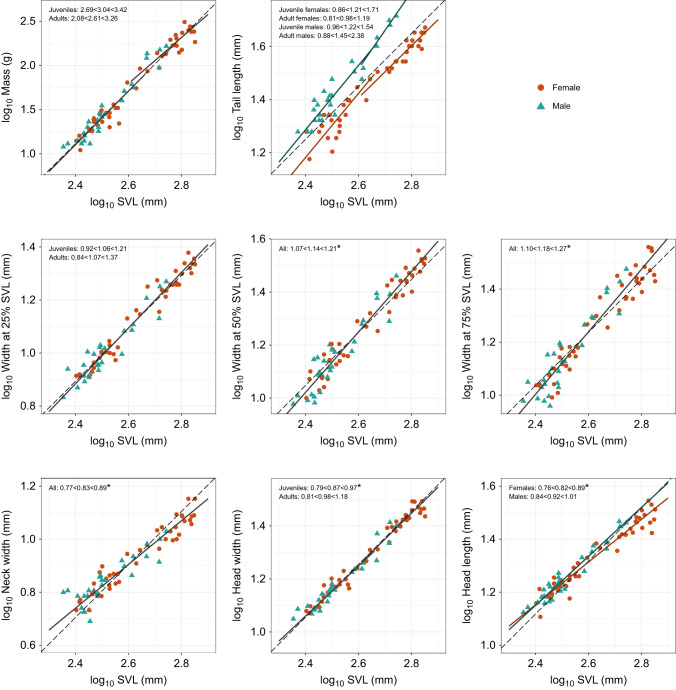
**Scaling of morphometric traits with snout–vent length (SVL) in sidewinder rattlesnakes, separated by sex and/or age class when appropriate.** Dashed lines have a slope equal to the expectation under isometry and pass through the mean value of (*x*,*y*) for all specimens. Solid lines represent reduced major axis (RMA) slopes for subgroups determined to be statistically distinct (Tables 3 and 4). Note that in many cases, the lines for distinct subgroups are quite similar. Estimated slopes along with 95% confidence intervals are labelled on plots. These plots and corresponding analyses do not include outliers (see Materials and Methods). Asterisks indicate traits that deviate significantly from isometry.

For scaling relationships, we focused on RMA results (Table 4, Fig. 3) because, unlike OLS, RMA does not make the assumption of zero measurement error in the independent variable and because RMA should minimize error when the relative error distributions are unknown (see Rayner, 1985 and references therein). OLS results are presented in Table S6. The scaling of most traits did not deviate significantly from isometry, including mass, tail length, width at 25% SVL and head width. Neck width scaled with negative allometry. Head length scaled with negative allometry in females, but with isometry in males. Width at 50% SVL and 75% SVL scaled with positive allometry. Ventral and subcaudal scale count had no significant relationship with SVL in either sex, so they were not included in the scaling analysis.

**Table 4.**
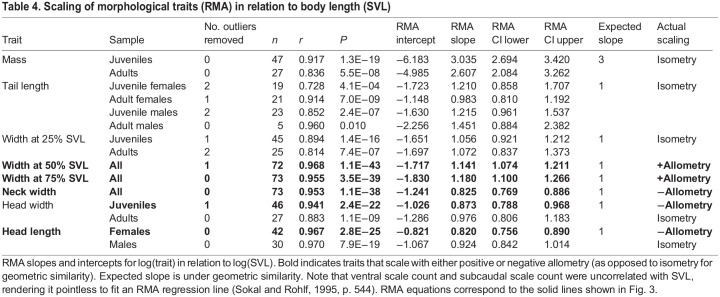
Scaling of morphological traits (RMA) in relation to body length (SVL)

The individuals in our study always moved using sidewinding locomotion, in line with previous observations of locomotor behavior in this species (Klauber, 1956). Although our statistical analysis included only 26 trials representing 26 individuals, we scored 197 trials representing 66 individuals for handedness, since sidewinding is an asymmetrical gait. Of these trials, 100 were left-handed, 96 were right-handed, and 1 started left-handed and then switched to right-handed. The individual that switched handedness mid-trial did so without stopping its motion, a behavior that is also performed periodically by sidewinders moving freely in the field (pers. obs.). Of the 62 individuals for which we scored three trials, 17 of them (27%) showed the same handedness for all trials (9 right-handed and 8 left-handed), approximately equal to the expectation if handedness were randomly selected for each trial (25%).

**Fig. 4.**
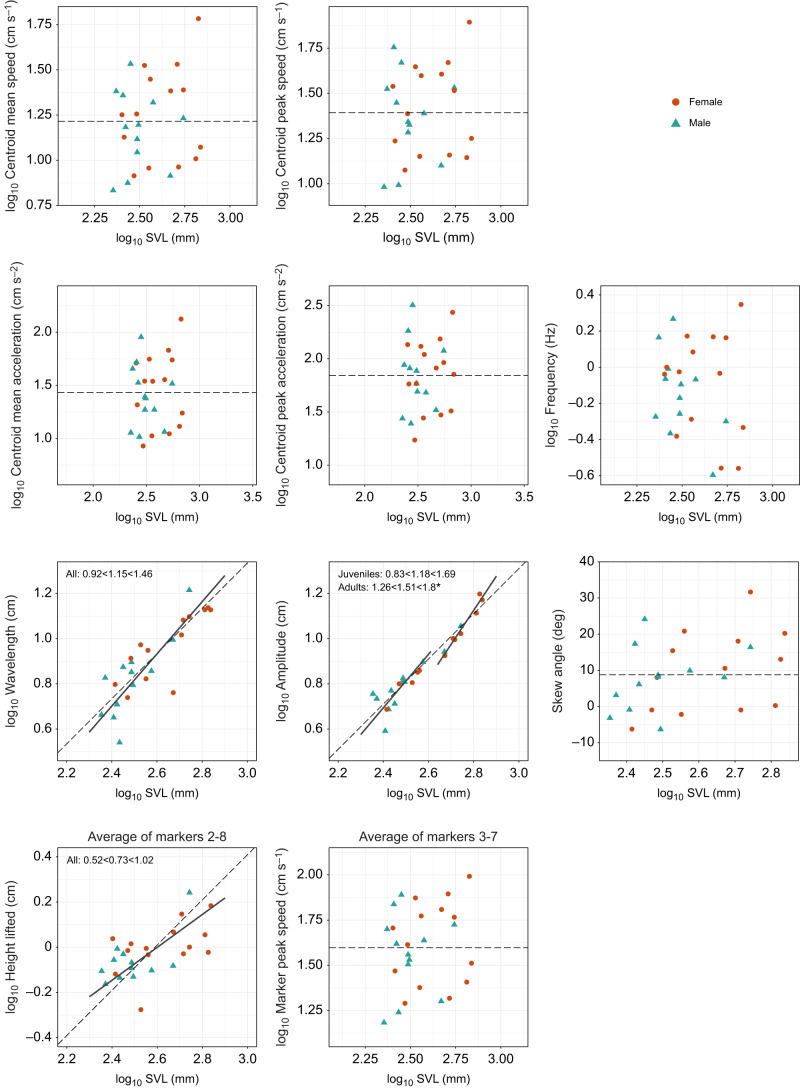
**Scaling of kinematic variables with snout–vent length (SVL) in sidewinder rattlesnakes, separated by age class when appropriate.** Dashed lines have a slope equal to the expectation under isometry (geometric similarity) and pass through the mean value of (*x*,*y*) for all specimens. Solid lines represent RMA slopes for subgroups determined to be statistically distinct (Tables 5 and 6). Estimated slopes along with 95% confidence intervals are labelled on plots that have solid lines representing RMAs. No outliers were identified for kinematic variables. Asterisks indicate variables that deviate significantly from isometry.

The best ANCOVA models showed that snake body temperature was not a significant predictor for any kinematic variable (Table 5). None of the kinematic variables showed sexual dimorphism. SVL was not a significant predictor of whole-snake speed (mean or peak), whole-snake acceleration (mean or peak), frequency, skew angle or the speed of individual markers. It was a significant predictor of amplitude, wavelength and height lifted. Additionally, age class and SVL had an interactive effect on amplitude, indicating different scaling relationships in juveniles versus adults. Wavelength and height lifted scaled isometrically (Table 6, Fig. 4; Table S6). Amplitude scaled with isometry in juveniles, but with positive allometry in adults (Table 6, Fig. 4).

**Table 5.**
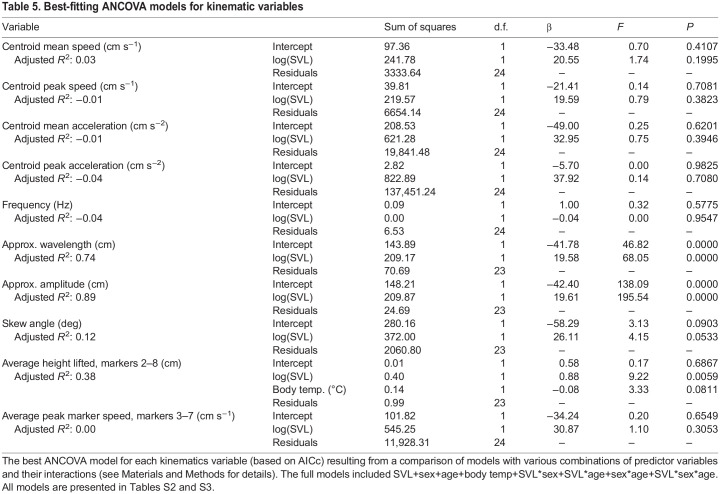
Best-fitting ANCOVA models for kinematic variables

**Table 6.**
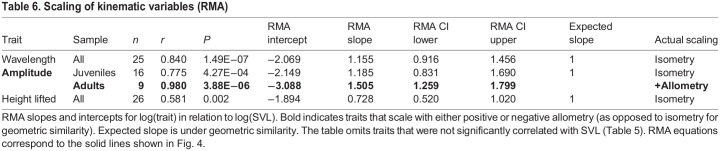
Scaling of kinematic variables (RMA)

Several kinematic variables were correlated with each other and with morphological variables (Table S4). Centroid mean and peak speed, centroid mean and peak acceleration, and peak speed of individual marker points were all highly correlated (*r*≥0.880, *P*≪0.001 for all pairs). Sidewinding frequency was highly correlated with each of these speed and acceleration metrics (*r*≥0.786, *P*≪0.001). Body width at 25% SVL was moderately correlated with speed and acceleration metrics (0.440≤*r*≤0.564, 0.002≤*P*≤0.025) and frequency (*r*=0.508, *P*=0.005). Body width at 50% SVL was correlated with wavelength (*r*=0.601).

Our preferred path model (Fig. 2) had no significant lack of fit (χ^2^=1.148, d.f.=2, *P*=0.563; RMSEA classic=0.0). Sidewinding frequency had the largest estimated effect on centroid mean speed (estimate±s.e.m.: 0.912±0.077; likelihood ratio test χ^2^=48.334, *P*<0.00001). Wavelength and amplitude had much smaller effects, and in opposite directions of each other (estimates±s.e.m.: 0.140±0.068 and −0.140±0.066, likelihood ratio test results χ^2^=3.946, *P*=0.047 and χ^2^=4.127, *P*=0.042, respectively). Skew angle did not have a significant effect on centroid mean speed (estimate±s.e.m.: −0.039±0.080; likelihood ratio test χ^2^=0.240, *P*=0.624), but it was positively correlated with frequency (estimate±s.e.m.: 0.548±0.223; likelihood ratio test χ^2^=9.301, *P*=0.002). Body width at 50% SVL had a positive effect on wavelength (estimate±s.e.m.: 0.588±0.159; likelihood ratio test χ^2^=10.808, *P*=0.001).

## DISCUSSION

Our study highlights several sources of intraspecific variation in the morphology and locomotor kinematics of sidewinder rattlesnakes. Several morphometric traits were sexually dimorphic and several scaled allometrically. However, we found no evidence of sexual dimorphism in sidewinding kinematics. The best ANCOVA models for kinematic variables indicated that speed, acceleration, frequency and skew angle did not vary with SVL, which underscores fundamental differences between a friction-dominated locomotor mode, such as sidewinding, and inertia-dominated modes, such as walking or running. RMA slopes indicated that linear dimensions characterizing the body wave largely scale isometrically; thus, wave shape generally does not change with body size. The exception was positive allometry of wave amplitude in adult sidewinders, an unexpected result. Finally, path analysis supported several hypothesized relationships among variables and revealed an unexpected correlation between skew angle and frequency (Fig. 2). All of these results are discussed in greater detail in the subsections below.

In addition to the study's main focus on intraspecific variation, scaling and morphology–kinematics–speed relationships, two additional results merit discussion. First, ectotherm locomotion is known to generally show strong temperature dependence (Bennett, 1990), yet we found no effects of temperature on any of the locomotor variables considered. The lack of temperature effects in our study may seem initially surprising, but temperature effects may be less evident in animals moving below their maximum performance capacity. We did not aim to elicit maximum performance because sidewinders generally use moderate speeds in the field (pers. obs.). Additionally, it is possible that the temperature range in our study (20.4–27.2°C for the substrate, 20.1–27.3°C for snake body temperature) did not include temperatures low enough or high enough for temperature effects to become evident.

Second, this study is the first to provide data on relative frequency of left- versus right-handed sidewinding, and we found no evidence for handedness. However, this result comes with the caveat that our study design was not optimal for determining whether sidewinders show handedness. Snakes were held in buckets prior to trials, then placed in the sandbox with snake tongs. Their initial choice of left- or right-handed sidewinding may have been influenced by placement in the sandbox and relative position of the experimenter or other objects, and trials were so short that they mainly captured initial choice and not handedness over long bouts of sidewinding. It would be interesting to examine locomotor trajectories in the field to determine the relative use of left- versus right-handed sidewinding and the frequency of switching between them. These data can conveniently be collected from tracks. Existing studies of handedness in appendage use (e.g. Bisazza et al., 1996; Papadatou-Pastou et al., 2020) and in locomotion via symmetrical gaits (e.g. Baciadonna et al., 2010; Buchanan et al., 2015) have established handedness as a phenomenon widespread across taxa. Asymmetrical gaits like sidewinding in snakes, sideways walking in crabs and bipedal galloping in sifakas provide opportunities for insight into handedness and motor control.

### Sexual dimorphism in morphometric traits

In our sample, adult female sidewinders were significantly larger than adult males, whereas juveniles showed no evidence of sexual size dimorphism. Female-biased size dimorphism has previously been documented in adult sidewinder rattlesnakes (Klauber, 1937, 1944) and in many other snake species (e.g. Hendry et al., 2014; Semlitsch and Gibbons, 1982; Shine, 1993, 1994) and is generally hypothesized to result from sex-specific natural selection, especially fecundity selection. Female sidewinders also had more ventral scales, which correspond 1:1 with trunk vertebrae (Alexander and Gans, 1966). Several studies of snakes have found sexual dimorphism in ventral scale (vertebral) count, often corresponding to sexual size dimorphism, sometimes female biased (e.g. Klauber, 1943; Lindell, 1996; Lindell et al., 1993; Shine, 2000) and sometimes male biased (e.g. Arnold, 1988; Arnold and Bennett, 1988; Dohm and Garland, 1993). A study of the European viper *Vipera berus* provided evidence for directional selection favoring individuals with higher ventral scale counts (Lindell et al., 1993). In our sample, juveniles had greater ventral count variance than did adults for females (Levene's test: *F*_1,41_=10.396, *P*=0.002) but not males (Levene's test: *F*_1,28_=2.040, *P*=0.164) and adult females had higher ventral scale counts than did juvenile females (Welch's *t*-test: *t*_29.353_=2.270, *P*=0.031), suggesting directional selection on ventral scale counts in females in this population.

Male sidewinders had longer tails and higher subcaudal scale counts than did females, in both juveniles and adults. Numerous studies have documented longer tails in males of many snake species, pointing out several possible explanations that are not mutually exclusive: the necessity of accommodating hemipenes at the base of the tail, selection on females to have a more posterior cloaca to maximize relative length of the body cavity and selection related to male behavior involving the tail during courtship (e.g. Kaufman and Gibbons, 1975; King, 1989; Klauber, 1943).

Males also had longer heads, relative to their body size. Because they swallow their prey whole, a snake's range of potential prey items is limited by its gape (Forsman and Lindell, 1993; Pough and Groves, 1983). If head length corresponds to underlying musculoskeletal traits that contribute to gape, then an increased relative head length could be a way for males to compensate for their smaller body size compared with females, expanding their otherwise restricted prey options. Indeed, some previous studies have found that longer heads enable snakes to more readily consume larger prey (e.g. Forsman and Lindell, 1993; Shine, 1991), although other studies have not supported this conclusion (e.g. Hampton, 2011; Vincent et al., 2006).

### Scaling of morphometric traits

Sidewinders of different sizes were not scale models: although most traits scaled with geometric similarity, some scaled allometrically. For example, body width measured at 50% SVL and at 75% SVL scaled with positive allometry, meaning that shorter sidewinders are relatively slender and longer sidewinders are relatively stout. Although it seems contradictory that body width scales allometrically while mass scales isometrically, this pair of results could suggest allometric scaling of other body dimensions that we did not measure, such as body height. It is unknown whether allometric scaling of external dimensions in our sample is indicative of allometric scaling of internal anatomy. If muscle cross-sectional area scales with positive allometry, for example, then it would likely have implications for locomotion. Scaling of muscle morphology could therefore be a fertile area for future study.

Head length scaled with negative allometry in females; thus, the head was disproportionately large in smaller individuals. Many studies have found evidence for negative allometry of head dimensions in snakes, both interspecifically (e.g. Tingle and Garland, 2021) and intraspecifically (e.g. Phillips and Shine, 2006; Vincent et al., 2006). Snakes are not unique in having relatively large heads at smaller sizes: scientists have long observed that vertebrate head size generally scales with negative allometry (Thompson, 1942, pp. 184–191). Negative allometry could have functional consequences for gape-limited predators like snakes. As noted above, snakes with longer heads may be able to consume larger prey (Forsman and Lindell, 1993; Shine, 1991), so negative allometry could allow smaller individuals to eat relatively larger meals.

### Scaling of sidewinding kinematics

In the absence (or considerable reduction) of postural costs and inertial forces, which strongly influence the scaling of morphology and locomotion in limbed animals, we hypothesized that the scaling of kinematics of sidewinding snakes would follow expectations derived from geometric similarity (Table 2). Consistent with this expectation, most kinematic variables did not deviate significantly from geometric similarity, with one exception: amplitude scaled with positive allometry in adult sidewinders (but not in juveniles), meaning that larger individuals had disproportionately large wave amplitude (Table 6). In general, deviations from geometric similarity often occur either because allometry serves as compensation to maintain functional equivalency, or because some size-dependent constraint prevents isometric scaling (Alexander, 2003).

Neither the existing literature nor the present dataset encourages us to speculate whether functional equivalency could explain the positive allometry of wave amplitude. Regarding the potential for size-dependent constraint, this situation often arises in locomotion owing to size effects on relative muscle force production. Under geometric similarity, muscle cross-sectional area and hence force-generating ability should scale as body length squared, whereas body mass scales as length cubed, so larger animals have reduced mass-specific force-generating ability. For muscular constraint to explain positive allometry of wave amplitude, higher-amplitude waves would have to require lower force production, which seems unlikely, so muscular constraint in larger sidewinders seems like an unlikely explanation for the pattern in our data. On the other hand, we did find positive allometry of body width, indicating stouter bodies in larger individuals. We do not know what changes in internal anatomy might underlie this trend; if it results from a disproportionate increase in muscle tissue, then it would invalidate our expectation of geometric scaling of kinematics. Here, it is worth noting that some species of lizards have positive allometry of thigh muscle mass, which may relate to their positive allometry of endurance capacity (or sprint speed, at least in some species) (Garland, 1984; Garland and Else, 1987). Directions for future studies include force measurements and examination of scaling and/or variation in muscle morphology, both of which would help us understand the mechanistic basis for kinematic differences in differently sized sidewinders.

Another possible explanation for the positive allometry of wave amplitude relates to the peculiarities of limbless terrestrial locomotion. The entire body can be used to generate ground reaction forces (as opposed to discrete limbs), affording limbless animals greater flexibility in how they use different sections of their bodies. Perhaps adult sidewinders use sections of their bodies differently as they get larger. For example, smaller snakes may use a greater percentage of the neck region to lift their heads high enough to see where they are going, removing that length from the total amount available for sidewinding. Additionally, snakes are not infinitely long, so they may also face trade-offs among wave amplitude, height lifted, number of waves present on the body (not measured in the present study) or other waveform parameters. Consider a finite length of string as a 2-dimensional analogy: if you lay the string on the table in the shape of a wave, and then increase the amplitude of the wave, you must also change either the wavelength or the number of wave cycles. Height lifted scales with a slope lower than 1 (Fig. 4), which might suggest that relatively higher amplitude in larger adults corresponds to a relative reduction in height lifted, but the slope is not statistically different from 1. Moreover, the magnitude of height lifted is so much smaller than that of amplitude that its contribution to body length usage would be trivial (see Table 2 for descriptive statistics; on average, amplitude was ∼8 times greater than height lifted). Therefore, we do not think a trade-off with height lifted explains why amplitude does not scale isometrically in adult sidewinders. Additionally, we did not find significant relationships between amplitude and any other variables in either the pairwise correlations or the path analysis (accounting for body size). Future work should consider additional parameters that we could not include, such as curvature, length or number of contact patches.

Our results for speed contrast with those of Secor et al. (1992), who found that speed increased with body size in sidewinder rattlesnakes. The prior study focused on maximal burst speed, unlike our study; therefore, even though sidewinders may become capable of higher speeds as they increase in size, they may not choose to move at higher speeds unless provoked by constant perturbation. It may also seem surprising that body size did not affect speed in our study given that both maximum and routine speed increase with size in many other species performing many types of locomotion (Cloyed et al., 2021), including other snakes (e.g. Arnold and Bennett, 1988; Garland, 1988). However, inertia-dominated locomotion must be faster in larger animals to maintain dynamic similarity (Alexander, 1991; Alexander and Jayes, 1983), whereas the friction-dominated locomotion does not face the same constraint because frictional force is independent of speed. Therefore, limbless terrestrial locomotors should have greater freedom to modulate their speed independently of body size.

### Relationships among morphology, kinematics and speed

The path analysis of residual (individual) variation supported four of our six hypothesized causal relationships among morphology, kinematics and speed (Fig. 2). First, it supported the positive relationship between body width and wavelength. Increasing wavelength without changing other parameters creates a wave with lower curvature. Thus, this relationship could plausibly exist because stouter snakes might not be able to curve their bodies as tightly as thinner snakes do.

Sidewinders in our study increased their speed mainly by increasing the frequency of sidewinding rather than stride length (distance moved per cycle) (Fig. 2). Frequency explained 83% of the variation in mean centroid speed, whereas wavelength and amplitude explained only about 2% each (based on squared path coefficients). This result aligns with that of Secor et al. (1992), who showed increasing frequency with increasing speed between 0.08 and 0.22 m s^−1^ (mean centroid speed in our study ranged from 0.07 to 0.61 m s^−1^). It makes sense that sidewinders would increase speed through changes in frequency rather than through wave parameters related to stride length, given that a sidewinding snake cannot increase its stride length beyond a certain point without reducing the number of body segments in contact with the ground. Sidewinding snakes normally maintain at least two contact points with the ground (Burdick et al., 1993; Jayne, 1988; Marvi et al., 2014); any fewer, and they lose stability while lifting their bodies, pivoting around their sole contact point (Jayne, 1988). Increasing frequency instead of stride length does not necessitate such a sacrifice in stability. This restriction on increased stride length in sidewinders contrasts with creatures from crawling maggots to galloping mice (or horses) to swimming fish, which can increase their speed by changing either stride length or frequency (or both simultaneously) (Bainbridge, 1958; Berrigan and Pepin, 1995; Heglund et al., 1974).

Skew angle varied considerably in the trials we analyzed, from −6.3 deg (a slight tail-wards tilt) to 31.7 deg (a strong head-wards tilt), and individuals can clearly modulate skew angle during sidewinding. Outside the trials analyzed, we observed that changes in the direction and magnitude of skew angle might coincide with acceleration and deceleration, but we could not examine this possibility in the present study because of our necessary focus on steady-state locomotion. We suspect that skew angle could play a role in controlling the direction of force vectors, an idea that could be tested in future studies.

Skew angle likely contributes to stride length in conjunction with wavelength, amplitude, and other wave parameters not captured in this study. We therefore expected that skew angle would be one of the variables affecting speed, given that speed equals stride length times frequency. Contrary to expectations, we found no relationship between skew angle and speed in the path analysis (Fig. 2). We did find that wavelength and amplitude predicted speed (positively and negatively, respectively), though they explained very little of the total variation in speed. Future studies could clarify the physical basis of stride length in sidewinders. In particular, a model that more fully describes the body's waveform could elucidate how various aspects of wave shape, including ones we could not measure here, contribute to stride length.

The relationship between skew angle and sidewinding frequency (Fig. 2) could have a physical and/or physiological basis, but our data do not allow us to explore that possibility. From a physical standpoint, mathematical relationships between skew angle and other variables are certainly complex and involve wave parameters that we did not characterize. From a physiological standpoint, increasing our knowledge of muscular mechanisms could clarify how sidewinders control skew angle and the consequences for ground reaction forces. Moreover, negative and positive skew angle are likely to be qualitatively different; we suspect they may involve different regions of muscle activation, in addition to different degrees of contraction of the same muscles. Therefore, it may not be appropriate to treat the entire range of sidewinder skew angle on a linear scale, but our sample size does not allow us to pursue more complicated schemes for scoring skew angle. Thus, future studies have much to explore with respect to the role of skew angle in sidewinding.

### Concluding remarks

This study is the first to examine the scaling of locomotor kinematics in a limbless terrestrial vertebrate, in which locomotion differs fundamentally from that of limbed terrestrial species owing to the reduction of postural costs and the dominance of friction over inertia. Therefore, most frameworks for understanding the scaling of limbed locomotion do not apply and geometric similarity provided a simple basis for our expectations. With one exception (wave amplitude in adults), we found that sidewinders of different sizes were geometrically similar in the shape of their waveform. Future studies of sidewinding and of additional limbless terrestrial gaits could move us towards a more general understanding of the principles behind their scaling, which matters because of the taxonomic and functional diversity of limbless terrestrial vertebrates, and because their locomotion has major applications for bioinspired design (e.g. Barazandeh et al., 2012; Chong et al., 2021; Fu et al., 2020; Gao et al., 2008; Hopkins et al., 2009; Li and Du, 2013; Liljebäck et al., 2012). Beyond scaling, we tested hypotheses regarding the relationships among morphology, kinematics, and speed, finding evidence for several of these relationships and laying the groundwork for future studies of kinematic variation in sidewinding species.

## Supplementary Material

Supplementary information
