# Supplementary material for: Scaling and relations of morphology with locomotor kinematics in the sidewinder rattlesnake Crotalus cerastes
Source: J Exp Biol. 2022 Apr 19;225(7):jeb243817. doi: 10.1242/jeb.243817 (PMC9080748; doi:10.1242/jeb.243817)
Supplement: Supplementary information [file jexbio-225-243817-s1.pdf]

Table S1. Comparison of various ANCOVA models for morphological traits
Spreadsheet containing a comparison of ANCOVA models for morphological traits. We started with a full model that included SVL + sex + age + SVL*sex + SVL*age + sex*age + SVL*sex*age, and we then eliminated predictor variables in a stepwise fashion, starting with interaction terms, until we had a model that included only SVL. We determined the best-fitting model for each trait based on AICc. Bold font indicates models with the lowest AICc for each trait. Orange highlighting indicates p values < 0.05, and light orange highlighting indicates p values between 0.05 and 0.1.

Click here to download Table S1

Table S2. Comparison of various ANCOVA models for kinematic variables (including body temperature as a predictor)
Spreadsheet containing a comparison of ANCOVA models for kinematic traits. We started with a full model that included SVL + sex + age + body temperature + SVL*sex + SVL*age + sex*age + SVL*sex*age, and we then eliminated predictor variables in a stepwise fashion, starting with interaction terms, until we had a model that included only SVL + body temperature. We determined the best-fitting model for each trait based on AICc. Gray font indicates that the best-fitting model for a given trait did not include body temperature, and is therefore presented in Table S3. Bold font indicates models with the lowest AICc for each trait. Orange highlighting indicates p values < 0.05, and light orange highlighting indicates p values between 0.05 and 0.1.

Click here to download Table S2

Table S3. Comparison of various ANCOVA models for kinematic variables (not including body temperature as a predictor)
Spreadsheet containing a comparison of ANCOVA models for kinematic traits. We started with a full model that included SVL + sex + age + SVL*sex + SVL*age + sex*age + SVL*sex*age, and we then eliminated predictor variables in a stepwise fashion, starting with interaction terms, until we had a model that included only SVL. We determined the best-fitting model for each trait based on AICc. Gray font indicates that the best-fitting model for a given trait included body temperature, and is therefore presented in Table S2. Bold font indicates models with the lowest AICc for each trait. Orange highlighting indicates p values < 0.05, and light orange highlighting indicates p values between 0.05 and 0.1.

Click here to download Table S3

Journal of Experimental Biology • Supplementary information

Table S4. Pearson correlation coefficients

Spreadsheet of Pearson correlation coefficients. We first log transformed all variables except for skew angle, which is signed, then regressed each variable on log SVL (including sex and/or age class as predictors in the regression if they appeared in the best ANCOVA model for a given variable), and finally used the residuals to compute correlation coefficients. Red font indicates significant correlations.

Click here to download Table S4

Table S5. Path analysis model comparison

Spreadsheet providing information on all path models that we considered. Our sample size limited us to models with only seven total variables. Given that we identified ten potential variables of interest, we compared models with different combinations of those variables. All models included hypothesized causal paths from frequency, wavelength, and peak-to-peak amplitude to mean centroid speed, because we had strong reason to think that those variables would show the clearest relationships. In addition to those four variables, the models included all possible combinations of tail length, ventral scale count, mass, and width at 50% SVL with their hypothesized effects on kinematics (except mass plus width at 50% SVL, which are redundant as measures of stoutness). We rejected nine of the 16 models because they had significant lack of fit based on a χ^2 lack-of-fit test. We compared the remaining models using root mean square error of approximation (RMSEA), which measures lack of fit per degree of freedom. Lower values indicate closer fit, with a lower bound of zero. Six models had RMSEA of zero. Of the variables included in those six models, skew angle and body width consistently had strong relationships with other variables, whereas height lifted and mass did not (models with vertebral count always showed significant lack of fit). Therefore, in the main text we present the model that includes skew angle, body width, and height lifted, which also had the lowest AICc of the six models with RMSEA of zero (Fig. 2). The “main take-aways” column presents the mean and standard error for all paths where the estimate was greater than the standard error. Bold text indicates paths where the estimate was greater than twice the standard error.

Click here to download Table S5

Journal of Experimental Biology • Supplementary information

Table S6. Scaling of morphological traits and kinematic variables (OLS)
Spreadsheet showing the results from OLS regressions of log(trait) on log(SVL). Bold indicates traits that scale with either positive or negative allometry (as opposed to isometry for geometric similarity).

Click here to download Table S6

Journal of Experimental Biology • Supplementary information
